# Eye-Tracking Metrics as a Digital Biomarker for Neurocognitive Disorders in Multiple Sclerosis: A Scoping Review

**DOI:** 10.3390/brainsci15020149

**Published:** 2025-01-31

**Authors:** Sonja Cecchetti, Andrew T. Duchowski, Marco Cavallo

**Affiliations:** 1 Department of Theoretical and Applied Sciences, eCampus University, 22060 Novedrate, Italy; cecchettisonja@gmail.com; 2 School of Computing, Clemson University, Clemson, SC 29634, USA; andrewd@cs.clemson.edu

**Keywords:** cognitive task, digital biomarker, eye movements, eye tracking, metrics, multiple sclerosis, neurodegenerative disorder, oculomotor system, saccades

## Abstract

Multiple sclerosis (MS) is an autoimmune disease classified as neurodegenerative because it can be associated with the more or less progressive development of neurological symptoms and cognitive deficits. In recent years, various studies have started to investigate eye movements in relation to cognitive impairment in persons with MS by means of eye-tracking equipment. However, the high heterogeneity of the paradigms used in different studies, as well as the different methodologies included, makes it difficult to provide a complete and precise picture of this important research and clinical issue. The purpose of the present in-depth scoping review was to map the existing literature in this field to determine which metrics may be relevant when dealing with the neurocognitive profile of people with MS. From the analyses of the included studies, the anti-saccade latency and errors were the most frequently proposed metrics. Correlation analyses between these metrics and cognitive measures showed significant associations between them, calling for a deeper investigation of this promising research and clinical field. The results of the present scoping review strongly suggest that eye tracking may play a crucial role in clinical practice during the early detection of neurocognitive disorders. There is a great need for primary research that addresses the full complexity of MS in its different phenotypes and the disease-related variables from a multidisciplinary perspective. Future research should clarify whether oculomotor dysfunction in MS follows or precedes cognitive deficits.

## 1. Introduction

### 1.1. Multiple Sclerosis

Multiple sclerosis (MS) was historically defined by Charcot [1] as a chronic and progressive disease of the central nervous system (CNS) that causes extensive focal lesions in the white matter of the brain and spinal cord, characterised by primary demyelination with variable degrees of axonal loss [2]. MS is an autoimmune disease classified as neurodegenerative because it can be associated with the more or less progressive development of neurological symptoms and cognitive–behavioural impairment. Onset typically occurs between the ages of 20 and 40, with a 3:1 female to male prevalence [3]. Epidemiological estimates suggest that there are over 2.8 million cases worldwide [4]. The aetiology is still unknown and the clinical course is highly variable, characterised by the sudden and acute onset of symptoms with an irregular pattern and timing [5].

Based on the clinical features, the following phenotypes can be categorised: clinically isolated syndrome (CIS), radiologically isolated syndrome (RIS), remitting relapsing multiple sclerosis (MS-RR), secondary progressive multiple sclerosis (SM-SP), primary progressive multiple sclerosis (SM-PP), and remitting progressive multiple sclerosis (SM-PR) [6,7]. Although MS is mainly associated with motor and sensory deficits, cognitive impairment is a common clinical feature, with prevalence ranging from 30% to 70% of cases [8,9]. The most common cognitive impairments relate to slowed processing speeds, attention, executive function, and episodic and working memory [10] and can affect all stages and phenotypes of MS, including RIS, its preclinical form [11,12].

In recent years, several researchers have been investigating eye movements in relation to cognitive impairment in persons with MS [13]. Eye movements are a fundamental aspect of vision and can be classified into two distinct classes of functions: those used to fixate the gaze and vestibulo-ocular and optokinetic movements and those used to change visual direction, vergence movements, slow pursuit movements, and saccades [14]. According to Pouget [15], eye movements are controlled by complex neural circuits connected to different brain networks, and alterations in the oculomotor system may indicate changes in the underlying neural circuits. Possible causes of impaired brain physiology include neurological diseases such as MS [16,17]. Oculomotor dysfunction is present in almost 90–95% of people with MS and is caused by the demyelination of the visual pathway [18,19]. In particular, demyelinating lesions of the posterior fossa may be the most common cause of oculomotor dysfunction [20].

### 1.2. Eye Tracking

Eye tracking is an innovative method for the investigation of gaze and eye movements associated with cognitive deficits. Classified as a computer and electronic device, the screen-based eye tracker is completely non-invasive. Commercially available eye trackers may differ technologically in their accuracy, precision, and sampling rate, which is why they exhibit differences in performance. Screen-based eye tracking, also known as video-oculography, uses infrared light to track the reflection of the cornea and the centre of the pupil [21]. This system requires, with each use, a calibration procedure, necessary to allow the eye tracker to calculate the eye position, and a subsequent validation procedure, before proceeding with task administration [22].

Experimental eye-tracking paradigms in MS can be divided into two main categories: oculomotor assessment tasks and visual cognitive assessment tasks. The most commonly used experimental paradigms in studies of people with MS are as follows.

- Extended fixation paradigm (also known as visual fixation task) [23]: Participants are instructed to maintain their gaze on a single point. Changes in fixation, such as frequent micro-saccades or instability, may indicate deficits in the areas of the brain responsible for visual stability.
- Pro-saccade paradigm (also known as visually guided saccade paradigm) [24]: Participants are instructed to quickly move their gaze to a target stimulus appearing at a specific position. Prosaccade paradigms can be reflexive, stimulus-driven, or voluntary, being more cognitively driven.
- Dynamic double-step paradigm [25]: Participants are instructed to quickly shift their gaze to a target stimulus. Before the target stimulus is reached, it will suddenly change position, requiring participants to adapt their movement in real time to reach the new target.
- Memory-guided saccade paradigm (also known as delayed saccade paradigm) [26]: Participants are instructed to memorise the position of the target and to move their eyes to it only when necessary.
- Anti-saccade paradigm [27]: Participants are instructed to look in the opposite direction to the target. Anti-saccadic movements require more inhibitory control, and people with MS often have difficulty with these tasks due to changes in the frontal area.
- Smooth pursuit paradigm [28]: Participants are instructed to follow a moving stimulus on the screen to assess the ability to maintain smooth fixation. Difficulties in following a target may indicate coordination problems and reflect lesions in the central nervous system.
- Spatial cueing paradigm (endogenous cues or exogenous cues) [29]: Participants are instructed to use the cue, a central signal (such as an arrow pointing in one direction), to voluntarily shift their attention to the indicated location when the target appears.
- Visual search paradigm [30]: Participants are instructed to find a specific object, a target, within a set of distractor objects. This task allows the assessment of selective visual attention and the processing speed, which are often slowed in patients.

From each task, it is possible to extract specific eye-tracking metrics that can be used to identify cognitive deficits, obtain differential diagnoses between different phenotypes, monitor disease progression, and evaluate the effectiveness of treatments. Metrics are measures that depend on the type of eye tracker used and can be multiple [31].

Some studies have shown that eye-tracking technology can detect eye movement alterations associated with MS [32], but there are also considerable methodological differences between the available studies and a lack of standardised protocols. Although the current scientific literature supports the usefulness of eye tracking as a potential digital biomarker in neurodegenerative conditions [14], the heterogeneity of the studies could be considered a limitation in both research and clinical practice. The purpose of this scoping review is to map the literature to understand which metrics may be more relevant when dealing with the neurocognitive profile of people with MS.

## 2. Materials and Methods

The present scoping review was conducted following the Joanna Briggs Institute (JBI) methodology for scoping reviews [33,34]. The Preferred Reporting Items for Systematic reviews and Meta-Analyses for Scoping Reviews (PRISMA-ScR) Checklist for reporting was used [35]. The scoping review protocol was registered in the Open Science Framework (OSF), PsyArXiv Preprints, in the Neuroscience section [36], on 18 October 2024 and is available online (https://osf.io/preprints/psyarxiv/92dth, accessed on 3 January 2025).

### 2.1. Research Team

To conduct a methodologically robust and clinically relevant scoping review, the research team consisted of authors with expertise in quantitative and qualitative research methodologies, evidence synthesis, cognitive neuroscience, clinical neuropsychology, MS, computer science, and eye tracking.

### 2.2. Review Question

The present work was guided by the following research question: ‘Which eye-tracking metrics could be considered most promising as a digital biomarker for neurocognitive disorders in multiple sclerosis?’

### 2.3. Elegibility Criteria

Only studies that met the following Population, Concept, and Context (PCC) criteria were included in the scoping review.

- Population. We included all studies that looked at any MS condition, with any diagnosis, limited to an adult population aged 18 years or older.
- Concept. We included all studies that investigated the relationship between neuropsychological assessment and screen-based eye tracking (infrared video-oculography).
- Context. This review considered studies conducted in any context.
- Types of evidence sources. This scoping review included any study design, published in English, in peer-reviewed indexed journals. No time, setting, or geographic restrictions were applied.

#### Exclusion Criteria

All studies that did not fulfil the specific criteria of the PCC (e.g., studies that had not used a screen-based eye tracker or studies that had not investigated the correlation between eye movements and the cognitive profile) were excluded.

### 2.4. Search Strategy

In this scoping review, a multi-stage search strategy was used. First, a limited PubMed search was conducted to identify articles on the topic. The terms in the titles, abstracts, and keywords used to describe the relevant articles were used to develop a comprehensive search strategy for the databases EBSCOhost, Cochrane Central, Scopus, and Web of Science, and for the registry ClinicalTrial.gov. Grey literature such as Google Scholar, the bibliographic references of each included study, and direct contact with experts in the fields of eye tracking and MS were also considered. The search strategy involved the use of specific keywords (such as ‘eye tracking’ and ‘multiple sclerosis’) and Boolean operators. The strings were adapted to each database used; a librarian was consulted for this purpose. In the Supplementary Materials (Table S1), all search strategies used are reported as per PRISMA-S (extension for reporting literature searches in systematic reviews) [37]. Searches were conducted until 2 November 2024 without date limitations.

### 2.5. Study Selection

**Figure 1:**
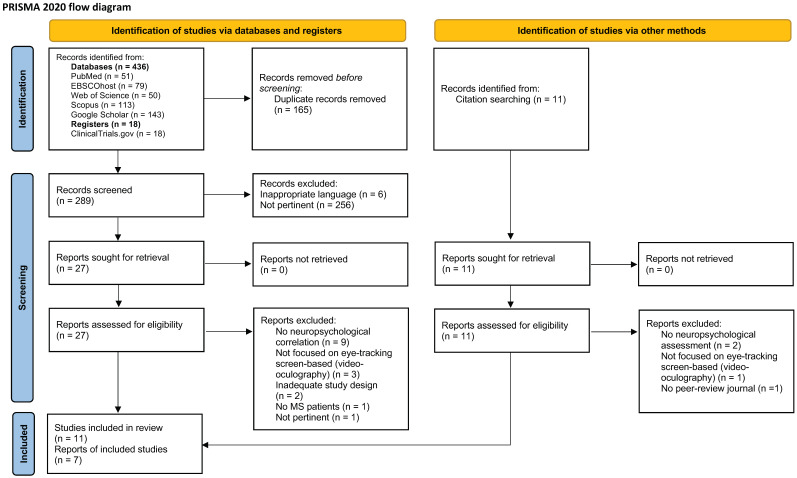
Preferred Reporting Items for Systematic Reviews and Meta-Analyses (PRISMA) 2020 flow diagram [40]. This work is licensed under CC BY 4.0. To view a copy of this license, visit https://creativecommons.org/licenses/by/4.0/, accessed on 15 November 2024.

Following the literature search, all bibliographic references were imported into EndNote 20/2020 [38] and duplicates removed. Two independent reviewers examined the titles and abstracts against the inclusion and exclusion criteria of the scoping review. For sources considered potentially relevant, all full texts were retrieved and subsequently imported into the JBI System for the Unified Management, Assessment and Review of Information (JBI, Adelaide, Australia) [39]. The two reviewers independently assessed all selected full texts according to the established criteria. Through discussion with a third reviewer, doubtful cases were resolved. The entire selection process was detailed using the PRISMA 2020 flow diagram [40] (Figure 1).

### 2.6. Data Extraction and Data Synthesis

Data extraction was carried out by two reviewers independently. The data of the selected articles were extracted using tools specially designed by the reviewers. Given the multitude of data, the initial draft of the tool was revised by the researchers to improve its accuracy. It was not possible to opt for a single table, but it was decided to report the extracted data in individual tables for the following categories: participants, neuropsychological assessment, eye-tracking equipment, metrics, and results. The extracted data were summarised in this scoping review in two complementary ways: numerically in tabular form and thematically through descriptive analysis. The synthesis of the data made it possible to answer the question of the scoping review and also offered further topics for discussion.

## 3. Results

From the PRISMA 2020 flow chart (Figure 1), the entire article selection process can be seen. From the initial literature search, 468 records were identified; following extensive screening, 450 were excluded and 18 articles were included [41,42,43,44,45,46,47,48,49,50,51,52,53,54,55,56,57,58].

### 3.1. Critical Appraisal of Included Studies

Although, in a scoping review, it is not essential to assess the quality of individual studies, we nevertheless decided to proceed with a critical appraisal to provide an overview of the literature by aggregating the results with greater methodological rigour. For this purpose, the JBI Critical Appraisal tool for use in JBI Systematic was applied [59]. All studies included were cross-sectional studies, except for one study [41], which was longitudinal. All studies included a control group, with the exception of one study [57]. Table 1, generated using the JBI SUMARI software [39], shows the quality assessment scores of each study using the Case Control Review Checklist [59]. The critical appraisal, conducted by two reviewers independently, showed high scores for all studies. Item 9, ‘Was the exposure period of interest long enough to be significant?’, was excluded from the evaluation because it was not applicable to the selected studies. The two reviewers reached complete agreement on the sources of disagreement through discussion.

### 3.2. Charateristics of Participants

The eighteen studies included 1463 participants, of whom 473 were healthy controls. Of the 990 participating patients, 75 were classified as having CIS-MS, 10 as RIS-MS, 514 as RR-MS, 125 as SP-MS, and 39 as PP-MS, while 99 had no specified phenotype. The majority of the MS sample was female: 631 females, 226 males, and 133 unspecified. The mean age of the participants, who were persons with MS, was 42.6 years (range 34.6–55.7). The characteristics of the participants in each study are given in the Supplementary Materials (Table S2).

### 3.3. Neuropsychological Assessment

The measures used to assess cognitive function were highly variable. Only five out of eighteen studies used a comprehensive MS-specific neuropsychological battery: two studies [48,57] used the Brief International Cognitive Assessment for MS (BICAMS) [60], two studies [54,56] used the Brief Repeatable Battery of Neuropsychological Tests (BRB-NT), and only one study [53] used the Minimal Assessment of Cognitive Function in MS (MACFIMS) [61]. Most studies used specific tests for the different cognitive domains. The most commonly used tests were the Paced Auditory Serial Addition Task (PASAT) [62] to assess attention, used in nine studies [41,42,43,44,45,46,47,50,53], and the SDMT in eleven studies [42,43,44,45,48,49,50,51,53,55,57]. The Symbol Digit Modalities Test (SDMT) [63] in particular is considered a useful sentinel test for screening for MS. Four studies [49,52,54,56] used the Stroop test to assess executive function. A descriptive table listing all of the neuropsychological, neuropsychiatric, and neurological tests administered in each study can be found in the Supplementary Materials (Table S3).

### 3.4. Eye-Tracking Equipment

In terms of the description of the equipment and setup used, there was considerable variation between the studies analysed and many data were missing. An overview can be found in Table S4 in the Supplementary Materials. Although only screen-based eye tracker models were considered, four studies [41,42,43,44] used the Skalar Medical IRIS infrared model, four studies [45,46,54,56] used various SR Research EyeLink models, and three studies [47,48,49] used various SMI Gmb models. Other studies employed eye tracker brands such as Tobii, Gazepoint, and SuriCog. The sampling frequency of the devices was between 30 Hz and 1000 Hz. Only eleven out of eighteen studies reported the spatial accuracy. This can have a value between 0 and 1; it should be noted that a lower value represents better accuracy.

With regard to the setup, only seven studies described the light conditions, and, of these, only two had planned to carry out the experiment in the dark [42,43]. It is useful to emphasise how important it is to avoid direct light from the windows, to have constant light conditions, and, better still, to work in the dark so as not to distort the outcomes. With regard to the eye movement recording system, three studies [45,46,52] opted for monocular signal recording, five [47,48,51,55,56] for binocular signal recording, and ten studies did not report this specification. Regarding head restraints, more than half—ten out of eighteen studies—reported using forehead or chin restraints. Five studies [51,53,54,55,56] reported having performed nine-point calibration, and only two studies [48,49] performed five-point calibration, while more than half—eleven studies—did not report these data. The most commonly used software for the creation of stimuli was E-Prime, used in four studies [41,42,43,44], and Experiment Builder, used in three [45,46,52]. MATLAB was the most commonly used software for the subsequent analysis of eye movements, observed in five studies [42,49,50,54,56].

### 3.5. Experimental Paradigms, Metrics, and Outcomes

For each study included in this scoping review, the researchers had planned to use one or more experimental screen-based eye-tracking paradigms. The most commonly used experimental tasks were the anti-saccade paradigm, used in eight studies [41,42,44,46,49,50,54,57]; the prosaccade paradigm, used in six studies [42,46,47,50,54,57]; the spatial cueing paradigm, used in three studies [42,46,52]; and the memory-guided saccade paradigm, used in only two studies [45,46]. In contrast, the following experimental tasks were only administered once: extended fixation paradigm [57], dynamic double-step paradigm [56], smooth pursuit paradigm [57], visual search paradigm [55], pupillometry paradigm [48], and recognition paradigm [58]. The choice of the experimental paradigm used to investigate the relationship between eye movements and neurocognitive disorders in people with MS is indicative of the importance of analysing saccadic movements.

For the pro-saccade paradigm, the most commonly analysed eye-tracking measures were saccade metrics, such as the saccade latency, saccade accuracy, and saccade gain. The saccade latency provided an indication of how quickly the participant had responded, the saccade accuracy provided an indication of how close they had come to the target, and the saccade gain showed how much distance had been covered from the target. For the anti-saccade paradigm, the most commonly analysed eye-tracking measures were various anti-saccade metrics, such as the latency error, corrected anti-saccade latency, correction time, error amplitude, corrected amplitude, final eye position, percentage of correct trials, and mean error latency.

In this scoping review, the most extracted metrics from the anti-saccade paradigm were the anti-saccade latency in eight studies [41,42,44,46,49,50,54,57] and the anti-saccade error in seven studies [41,44,46,49,50,52,54].

Correlation analyses between these metrics and various neuropsychological tests showed that two studies [41,42] found a significant correlation between the PASAT scores and AS errors. However, another study [44] found a significant inverse correlation between the PASAT scores and AS errors and between the SDMT scores and AS errors. Lower scores on the neuropsychological tests correlated with a higher number of AS errors. No significant correlations were found between the cognitive variables and AS latency in this study. In another study [46], lower PASAT scores were associated with more AS errors and longer AS latency. In [49], no statistically significant correlations were found between the different neuropsychological tests and the AS latency and errors. In contrast to the previous study, the authors of [50] found a correlation between the AS latency and PASAT score. In [52], statistical analyses showed that the group of people with MS exhibited more AS errors and had a lower interference ratio score on the Stroop test. In [54], statistical analyses found significant correlations between the saccadic latency and lower scores in executive function, working memory, and information processing speed.

A summary of all results from the studies included in this scoping review can be found in the Supplementary Materials (Table S5).

**Figure 2:**
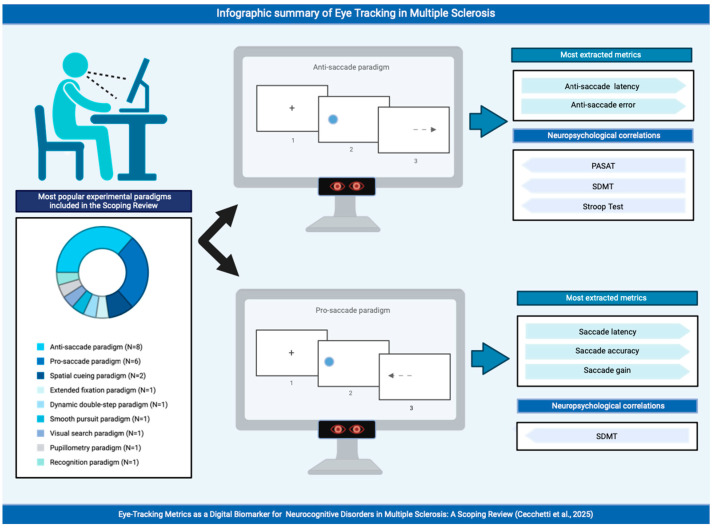
Infographic summary of eye tracking in multiple sclerosis. Created in BioRender. Cecchetti, S. (2025), https://BioRender.com/i19d327, accessed on 23 January 2025.

In addition, Figure 2 presents an infographic summarising the most common methodological aspects of the studies here reviewed.

## 4. Discussion

This scoping review of the literature on MS and eye tracking identified a lack of longitudinal studies and research designs that included participants with different MS phenotypes in their samples.

With regard to neuropsychological assessment, it would be appropriate to assess different cognitive domains by administering a comprehensive MS-specific battery, such as the MACFIMS or the BRB-NT [64,65]. An analysis of the data showed that only four studies had used comprehensive batteries for MS-specific neuropsychological assessment. Unfortunately, these batteries require time and neuropsychological expertise, but they could provide a better understanding of the relationship between eye movements and cognitive deficits. It should be noted that the cognitive domains most affected in MS may relate to processing speeds, long-term memory, working memory, attention, visuospatial perception, and additional components of the executive function domain [66]. As the most common pattern of cognitive deficits in MS has been defined as “fronto-subcortical syndrome” (even if other structures are implied too), it would therefore be appropriate to assess in detail the executive functions corresponding to the frontal neuroanatomical areas potentially affected in MS [67]. In addition to administering the BRB-NT battery, it would thus be necessary to integrate the neuropsychological assessment with other tests specifically tailored to executive function [68], such as the Stroop test, the FAB, or the DKEFS. Four studies included in this scoping review administered the Stroop test, as highlighted in the results.

In most studies (12 out of 18), the EDSS disability scale was administered by a neurologist, although the literature suggests that there are no particularly significant correlations between the EDSS scale and cognitive impairment [67].

As far as neuropsychiatric disorders are concerned, the literature reports that a high percentage, approximately 50% of patients, are affected by depressive disorders throughout their lives [69,70]. We know that psychiatric disorders affect eye movements [71], and, in this regard, the BDI scale was administered in seven studies to homogenise the samples by excluding depressive disorders. It should be made clear that depressive disorders in MS are bidirectionally related to cognitive disorders [67]. Mood disorders may influence cognitive disorders, but cognitive disorders may also cause mood disorders, and both may correlate with eye and gaze movements. To this end, it would be interesting to apply regression-based mediation and moderation statistical approaches in future clinical research [72].

It is important to note that the studies included in this scoping review were published over a long period of time. Therefore, given the exponential technological development in this field, some of the eye trackers mentioned are no longer available on the market and new or even better-performing models have been introduced. Looking at the eye trackers currently on the market and our experience in the field, we could consider the EyeLink Portable Duo from SR Research as the ‘gold standard’ in terms of reliability, mainly due to its fast sampling rate of up to 2000 Hz; the Tobii Pro Spectrum is also good, with up to 1200 Hz; and the GP3 Gazepoint has a maximum frequency of 150 Hz, being slower but more affordable. These considerations are supported by over 12,000 citations in scientific publications for both SR Research and Tobii.

The question remains as to the sampling frequency that an eye tracker should have and whether there is a minimum Hz threshold below which it should not fall. We have tried to address this issue, but the answer depends on the research question. According to the Nyquist–Shannon theorem [73], the sampling rate should be exactly two times the frequency to be detected. This value in Nyquist’s theorem is intended to ensure that the recorded signal can be reproduced without loss, as there should be no aliasing artefacts at twice the speed. We must remember that each eye movement has different characteristics and therefore the minimum required value of the sampling rate could vary depending on the experimental paradigm that we perform and the metrics that we decide to extract [74].

Concerning eye-tracking procedures, the lack of standardised protocols, the use of different eye trackers with different characteristics and performance, and the different setup conditions are highlighted. According to the most recent guidelines [75], a very important methodological aspect of an eye-tracking study is the need to report all procedures that were performed, describing the equipment and the setup, in order to facilitate the reader’s understanding and the reproducibility of the work. Without the correct specifications, the parameters could vary, leading to other researchers obtaining different results for the same research question [76].

In this context, it is worth noting that experiments can be created using a variety of software. Some of these, which were also used in the studies included in the scoping review but require payment for a licence, are Experiment Builder, Tobii Pro Lab, and Gazepoint Analysis. A good alternative that we suggest is PsychoPy [77], an open-source, easy-to-use software program that allows the integration of several physiological measures at the same time. Furthermore, the code could be shared on platforms (such as GitHub), which would allow the study to be replicated by other researchers using different eye trackers.

In terms of experimental eye-tracking paradigms, the saccadic paradigm was the most commonly used in the studies included in this scoping review. Most authors seem to agree that the analysis of saccadic eye movements plays a key role in MS. Indeed, saccadic dysfunction, such as saccadic dysmetria and altered regular pursuit, is common in people with MS [78]. The metrics that showed the most promise as digital biomarkers were the AS latency and AS errors. It is important to note that these metrics can be influenced by the target shape, presentation time, and other factors, making it difficult to compare the results across studies [79].

Considering the anti-saccade paradigm, according to the Nyquist–Shannon theorem, if the average anti-saccade latency is about 250 ms, the minimum frequency would be about 500 Hz, but higher sampling frequencies would ensure better accuracy. Theoretically, one could assume that 100–250 Hz would be sufficient for behavioural studies, whereas >500 Hz would be more suitable for neurophysiological studies. For the studies included in this scoping review that used the anti-saccade paradigm, most of the eye trackers used had a high sampling frequency of 1000 Hz. The only two studies [49,57] that used 60 Hz devices showed different results. Given the lack of studies involving people with MS, it would be useful to design comparative studies of eye trackers by sharing the code between researchers.

Although correlation analyses between the anti-saccade paradigm metrics and cognitive variables have yielded mixed results, the majority of studies have shown statistically significant correlations between cognitive tests, such as the PASAT, SDMT, and Stroop test, and eye-tracking metrics, such as the AS latency and AS errors. Some studies [41,42,44,46] found a correlation between the PASAT and AS errors; others [46,50] found a correlation between the PASAT and AS latency. The PASAT is a test of sustained attention and concentration and is particularly useful because it does not involve visuomotor skills. The study in [44] found a correlation between the SDMT and the AS error, while another [54] found a correlation between the SDMT and the AS latency. The SDMT is a test of the information processing speed and working memory; it is a test on which people with MS perform below the norm. Its use as a screening test has been suggested because it is one of the first tests on which patients show low scores [80].

The capacity for social cognition has only recently been investigated in people with MS, and, according to a meta-analysis [81], patients show deficits in both theory of mind tasks and facial emotion recognition, with greater difficulty in recognising negative emotions. Theory of mind tasks and facial emotion recognition were found to correlate with executive function, working memory, and processing speeds. In line with these findings, one study included in this scoping review [58] investigated facial emotion recognition (FER) using eye tracking. The results showed that 21% of participants with MS had significant impairments in FER, with different pathways being scanned depending on the MS phenotype.

Recently, some researchers [82] have suggested using pupil dilation as a metric to investigate its association with cognitive fatigue, but the relevant study [48] included in this scoping review did not show statistically significant differences in people with MS.

In terms of future directions, it remains to be clarified whether oculomotor dysfunction in MS follows or precedes cognitive deficits.

The new criteria proposed by McDonald in 2024 continue to rely on the use of MRI as the main diagnostic tool, but they place greater emphasis on the integration of clinical data and other imaging modalities to aid in differential diagnosis, especially in the early stages of the disease. In this context, eye tracking is emerging as an innovative and promising technology to objectively analyse gaze and eye movements. People with MS with cognitive impairment report poorer quality of life and greater difficulty in maintaining work activities [83]. Several systematic reviews [84,85] have found inconclusive results regarding the efficacy of the pharmacological treatment of cognitive impairment, but, according to other literature reviews [86,87], cognitive rehabilitation would be useful for people with MS. In this context, eye tracking could facilitate the diagnosis of MS at an early stage, allowing people with MS to start rehabilitation at the opportune time. Therefore, it is considered crucial to approach the field of eye tracking from an open science perspective, as it is only through the replicability and reproducibility of studies that the results can be generalised.

### 4.1. Research Implications and Suggestions for Clinical Practice

Based on the collected scientific evidence, we support the view that the next decade of clinical research will likely lead to the inclusion of eye-tracking metrics in clinical cognitive testing [88,89]. Thanks to the current technological developments and the implementation of eye tracker research via webcams [90], we can assume that, in the near future, eye tracking will be integrated into remote neuropsychological assessments, just as Cavallo et al. [91] argued that online therapy will become an integral part of current clinical practice. In addition, eye tracking could be useful for patients not only as a diagnostic aid but also for rehabilitation. Therefore, there is a need to provide the scientific community with more detailed information on gaze and eye movement metrics obtained from experimental paradigms involving patients. With this scoping review, the authors hope that the evidence gathered will stimulate the interest of clinicians and encourage innovative interdisciplinary collaborations for further research in this area.

### 4.2. Strengths and Limitations

Although we assessed the methodological quality of the individual studies included in this scoping review, the heterogeneity of the experimental paradigms and procedures made it impossible to perform a systematic review and meta-analysis to draw conclusions about the most effective eye-tracking metrics. Our scoping review is, to the best of our knowledge, the first study to address this research question. We reviewed the literature and identified the most promising metrics as potential digital biomarkers for MS. We also mapped key concepts and identified the next research priorities.

## 5. Conclusions

Here, we discussed the wide variety of tasks proposed and which metrics might be most promising for early diagnosis and monitoring in patients. The anti-saccade latency and AS errors were the most frequently proposed metrics. The results of the present work demonstrate the potential of the eye-tracking method for integration into clinical practice in the detection of neurocognitive disorders. At the same time, this is a vast and complex field that requires interdisciplinary and synergistic work between several disciplines: neurology, visual science, neuropsychology, neuroscience, computer science, and bioengineering. Therefore, there is a great need for primary research that addresses the full complexity of MS in its different phenotypes and the disease-related variables from a multidisciplinary perspective.

## Figures and Tables

**Table 1:** Quality assessment—JBI Case Control Study Checklist.

Authors, Published Year	1	2	3	4	5	6	7	8	9	10	Total Score
Fielding et al., 2009 [42]	Y	Y	Y	Y	Y	Y	Y	Y	-	Y	**Y 9/9** **N 0/9** **U 0/9**
Fielding et al., 2009 [43]	Y	Y	Y	Y	Y	Y	Y	Y	-	Y	**Y 9/9** **N 0/9** **U 0/9**
Fielding et al., 2012 [41]	Y	Y	Y	Y	Y	Y	Y	Y	-	Y	**Y 9/9** **N 0/9** **U 0/9**
Kolbe et al., 2014 [44]	Y	Y	Y	Y	Y	Y	Y	Y	-	Y	**Y 9/9** **N 0/9** **U 0/9**
Clough et al., 2015 [45]	Y	Y	Y	Y	Y	Y	Y	Y	-	Y	**Y 9/9** **N 0/9** **U 0/9**
Clough et al., 2015 [46]	Y	Y	Y	Y	Y	Y	Y	Y	-	Y	**Y 9/9** **N 0/9** **U 0/9**
Nygaard et al., 2015 [47]	Y	Y	Y	Y	Y	Y	Y	Y	-	Y	**Y 9/9** **N 0/9** **U 0/9**
de Rodez Benavent et al., 2017 [48]	Y	Y	Y	Y	Y	Y	Y	Y	-	Y	**Y 9/9** **N 0/9** **U 0/9**
Ferreira et al., 2018 [49]	Y	Y	Y	Y	Y	Y	Y	Y	-	Y	**Y 9/9** **N 0/9** **U 0/9**
Gajamange et al., 2019 [50]	Y	Y	U	Y	Y	Y	Y	Y	-	Y	**Y 8/9** **N 0/9** **U 1/9**
Pavisian et al., 2019 [51]	Y	Y	Y	Y	Y	Y	Y	Y	-	Y	**Y 9/9** **N 0/9** **U 0/9**
Ternes et al., 2019 [52]	Y	Y	Y	Y	Y	Y	Y	Y	-	Y	**Y 9/9** **N 0/9** **U 0/9**
Zangemeister et al., 2020 [53]	U	U	Y	Y	Y	Y	Y	U	-	Y	**Y 7/9** **N 0/9** **U 3/9**
Nij Bijvank et al., 2021 [54]	Y	Y	U	Y	Y	Y	Y	Y	-	Y	**Y 8/9** **N 0/9** **U 1/9**
Gehrig et al., 2022 [55]	U	U	U	Y	Y	Y	Y	U	-	Y	**Y 6/9** **N 0/9** **U 4/9**
Nij Bijvank et al., 2023 [56]	Y	Y	U	Y	Y	Y	Y	Y	-	Y	**Y 8/9** **N 0/9** **U 1/9**
de Villers-Sidani et al., 2023 [57]	N	N	Y	Y	Y	Y	Y	N	-	Y	**Y 7/9** **N 3/9** **U 0/9**
Polet et al., 2023 [58]	Y	Y	Y	Y	Y	Y	Y	Y	-	Y	**Y 9/9** **N 0/9** **U 0/9**
**Total item score**	**Y 15/18** **N 1/18** **U 2/18**	**Y 15/18** **N 1/18** **U 2/18**	**Y 14/18** **N 0/18** **U 4/18**	**Y 18/18** **N 0/18** **U 0/18**	**Y 18/18** **N 0/18** **U 0/18**	**Y 18/18** **N 0/18** **U 0/18**	**Y 18/18** **N 0/18** **U 0/18**	**Y 15/18** **N 1/18** **U 2/18**	**Y 0/18** **N 0/18** **U 0/18**	**Y 18/18** **N 0/18** **U 0/18**	

Scoring of Items: Y = yes, N = no, U = unclear, - = N/A. Checklist Items [59]: 1. Were the groups comparable other than the presence of disease in cases or the absence of disease in controls? 2. Were cases and controls matched appropriately? 3. Were the same criteria used for the identification of cases and controls? 4. Was exposure measured in a standard, valid, and reliable way? 5. Was exposure measured in the same way for cases and controls? 6. Were confounding factors identified? 7. Were strategies to deal with confounding factors stated? 8. Were outcomes assessed in a standard, valid, and reliable way for cases and controls? 9. Was the exposure period of interest long enough to be meaningful? 10. Was appropriate statistical analysis used?

## Data Availability

The data that support the findings of this study are available in the Supplementary Materials.

## Supplementary Materials

The following supporting information can be downloaded at: https://www.mdpi.com/article/10.3390/brainsci15020149/s1, Table S1. Search strategy for each database/registry; Table S2. Participants’ characteristics; Table S3. Neuropsychological, neuropsychiatric and neurological assessment; Table S4. Eye-Tracking set up; Table S5. Experimental paradigms, metrics and results.
