# Supplementary material for: Eye-Tracking Metrics as a Digital Biomarker for Neurocognitive Disorders in Multiple Sclerosis: A Scoping Review
Source: Brain Sci. 2025 Jan 31;15(2):149. doi: 10.3390/brainsci15020149 (PMC11852410; doi:10.3390/brainsci15020149)
Supplement: Supplementary file 1 [file brainsci-15-00149-s001.zip › Table S1. Search strategy for each database_registry.docx]

| Table S1. Search strategy for each database/registry | |  |
| --- | --- | --- |
| **Database/Registry** | **Search strategy** | **Results** |
| PubMed | "multiple sclerosis"[All Fields] AND "eye track*"[All Fields] | 2/11/24 🡪 51 |
| EBSCOhost | ("multiple sclerosis" and "eye track*") Proximity, NO LIMITS. | 2/11/24 🡪79 |
| Web of Science | ("multiple sclerosis" and "eye track*") (All Fields) | 2/11/24 🡪 50 |
| Scopus | Article title, Abstract, Keywords ("multiple sclerosis" AND "eye track*") | 2/11/24 🡪113 |
| Cochrane Library | (“eye track*” OR “eye move*” OR “oculomot*” OR pupil*” OR “eye fixa*” OR “eye saccad*”AND "multiple sclerosis" OR MS) in All Text | 2/11/24 🡪 0 |
| Google Scholar | ("multiple sclerosis" and "eye track*") | 2/11/24 🡪143 |
| ClinicalTrials.gov | [Condition/disease] (multiple sclerosis) AND [other terms] (eye-tracking) | 2/11/24 🡪18 |
| Search date: November 2, 2024. No date and language restrictions. TOTAL 454 articles. | | |
