# Supplementary material for: Eye-Tracking Metrics as a Digital Biomarker for Neurocognitive Disorders in Multiple Sclerosis: A Scoping Review
Source: Brain Sci. 2025 Jan 31;15(2):149. doi: 10.3390/brainsci15020149 (PMC11852410; doi:10.3390/brainsci15020149)
Supplement: Supplementary file 1 [file brainsci-15-00149-s001.zip › Table S2. Participants' characteristics.docx]

| \|  \| Table S2. Participants’ characteristics \| \| \| \| \| \| \|  \|  \|  \| \| --- \| --- \| --- \| --- \| --- \| --- \| --- \| --- \| --- \| --- \| --- \| \| **Authors, Published Year** \| **MS Group** \| **CIS-MS** \| **RIS-MS** \| **RR-MS** \| **SP-MS** \| **PP-MS** \| **Other** \| **Mean Age (SD or Range)** \| **Gender (M/F)** \| **HC Group (Control)** \| \| Fielding et al., 2009 \| n = 25 \|  \|  \| n = 22 \| n = 3 \|  \|  \| 39 (24–58) \| N/A \| n = 25 \| \| Fielding et al., 2009 \| n = 25 \|  \|  \|  \|  \|  \|  \| 39.72 (24–58) \| (4/21) \| n = 25 \| \| Fielding et al., 2012 \| n = 24 \|  \|  \| n = 24 \|  \|  \|  \| 41 (26-56) \| N/A \| n = 12 \| \| Kolbe et al., 2014 \| n = 24 \|  \|  \| n = 24 \|  \|  \|  \| 47 (28-63) \| N/A \| n = 14 \| \| Clough et al., 2015 \| n = 66 \| n = 22 \|  \|  \|  \|  \| n = 22 CD-early-MS n = 22 CD-late-MS \| 35.45 ± 8.57 40.86 ± 11.14 47.36 ± 9.99 \| (5/17) (4/18) (0/22) \| n = 22 \| \| Clough et al., 2015 \| n = 74 \| n = 25 \|  \|  \|  \|  \| n = 25 CD-early-MS n = 24 CD-late-MS \| 34.6 ± 8.25 40.22 ± 11.62 47.21 ± 9.65 \| (5/20) (4/21) (0/24) \| n = 25 \| \| Nygaard et al., 2015 \| n = 44 \|  \|  \| n = 44 \|  \|  \|  \| 35.1 ± 7.3 \| (12/32) \| n = 41 \| \| de Rodez Benavent et al., 2017 \| n = 41 \|  \|  \| n = 41 \|  \|  \|  \| 35 ± 7.4 \| (13/28) \| n = 43 \| \| Ferreira et al., 2018 \| n = 38 \|  \|  \|  \|  \|  \|  \| 37 ± 6 \| (14/24) \| n = 38 \| \| Gajamange et al., 2019 \| n = 18 \| n = 18 \|  \|  \|  \|  \|  \| 36.50 ± 10.19 \| (3/15) \| n = 17 \| \| Pavisian et al., 2019 \| n = 33 \|  \|  \|  \|  \|  \|  \| 41.42 ± 9.89 \| (11/22) \| n = 25 \| \| Ternes et al., 2019 \| n = 41 \|  \|  \| n = 41 \|  \|  \|  \| 42.34 ± 9.45 \| (5/36) \| n = 25 \| \| Zangemeister et al., 2020 \| n = 28* \|  \|  \|  \|  \|  \|  \| 41.7 ± 1.4 \| (2/26) \| n = 21** \| \| Nij Bijvank et al., 2021 \| n = 176 \|  \|  \| n = 115 \| n = 47 \| n = 11 \| n = 3 Unspecified-MS \| 54.0 ± 10.8 \| (55/121) \| n = 33 \| \| Gehrig et al., 2022 \| n = 12° \|  \|  \| n = 12 \|  \|  \|  \| 42.50 ± 11.33 \| (5/7) \| n = 24°° \| \| Nij Bijvank et al., 2023 \| n = 209 \|  \|  \| n = 130 \| n =54 \| n = 18 \| n = 3 Unspecified-MS \| 54.3 ± 10.5 \| (67/142) \| n = 60 \| \| de Villers-Sidani et al., 2023 \| n = 60 \|  \|  \| n = 49 \| n = 11 \|  \|  \| 51.0 ± 10.6 \| N/A \| N/A \| \| Polet et al., 2023 \| n = 52 \| n = 10 \| n = 10 \| n = 12 \| n = 10 \| n = 10 \|  \| 40.4 ± 9.9 41 ± 11 44.2 ± 7 55.7 ± 6.5 53.8 ± 11.9 \| (0/10) (2/8) (5/7) (4/6) (6/4) \| n = 23 \| \| N/A = Not Available; *MS-fatigue; **MS-not fatigue; °MS-impaired; °°MS-not impaired; \| \| \| \| \| \| \| \| \| \| \| |
| --- | --- | --- | --- | --- | --- | --- | --- | --- | --- | --- | --- | --- | --- | --- | --- | --- | --- | --- | --- | --- | --- | --- | --- | --- | --- | --- | --- | --- | --- | --- | --- | --- | --- | --- | --- | --- | --- | --- | --- | --- | --- | --- | --- | --- | --- | --- | --- | --- | --- | --- | --- | --- | --- | --- | --- | --- | --- | --- | --- | --- | --- | --- | --- | --- | --- | --- | --- | --- | --- | --- | --- | --- | --- | --- | --- | --- | --- | --- | --- | --- | --- | --- | --- | --- | --- | --- | --- | --- | --- | --- | --- | --- | --- | --- | --- | --- | --- | --- | --- | --- | --- | --- | --- | --- | --- | --- | --- | --- | --- | --- | --- | --- | --- | --- | --- | --- | --- | --- | --- | --- | --- | --- | --- | --- | --- | --- | --- | --- | --- | --- | --- | --- | --- | --- | --- | --- | --- | --- | --- | --- | --- | --- | --- | --- | --- | --- | --- | --- | --- | --- | --- | --- | --- | --- | --- | --- | --- | --- | --- | --- | --- | --- | --- | --- | --- | --- | --- | --- | --- | --- | --- | --- | --- | --- | --- | --- | --- | --- | --- | --- | --- | --- | --- | --- | --- | --- | --- | --- | --- | --- | --- | --- | --- | --- | --- | --- | --- | --- | --- | --- | --- | --- | --- | --- | --- | --- | --- | --- | --- | --- | --- | --- | --- | --- | --- | --- | --- | --- | --- | --- | --- | --- | --- | --- | --- | --- | --- | --- | --- | --- | --- |
