# Supplementary material for: Eye-Tracking Metrics as a Digital Biomarker for Neurocognitive Disorders in Multiple Sclerosis: A Scoping Review
Source: Brain Sci. 2025 Jan 31;15(2):149. doi: 10.3390/brainsci15020149 (PMC11852410; doi:10.3390/brainsci15020149)
Supplement: Supplementary file 1 [file brainsci-15-00149-s001.zip › Table S3. Neuropsychological, neuropsychiatric and neurological assessment.docx]

|  | Table S3. Neuropsychological, neuropsychiatric and neurological assessment | | | | | | | | | | | | | | | | | | | |  |  |  |  |  |  |  |  |  |  |  |
| --- | --- | --- | --- | --- | --- | --- | --- | --- | --- | --- | --- | --- | --- | --- | --- | --- | --- | --- | --- | --- | --- | --- | --- | --- | --- | --- | --- | --- | --- | --- | --- |
| **Authors, Published Year** | **MOCA** | **MACFIMS** | **BICAMS** | **BRB-NT** | **SDMT** | **PASAT** | **RAVLT** | **DIGIT SPAN FORW.** | **DIGIT SPAN BACK.** | **CVLT** | **STROOP** | **BENTON FRT** | **BVMT** | **TRAIL MAKING** | **COWAT** | **DKEFS 20Q** | **TAP** | **CONCEPT SHIFTING TEST** | **MEMORY COMPARISON TEST** | **BEC-96** | **BELLS TEST** | **ORAL DENOMINATION IMAGES** | **HADS** | **BDI** | **HAQUAMS** | **WTAR** | **NART** | **MFIS** | **FSS** | **9HP** | **EDSS** |
| Fielding et al., 2009 |  |  |  |  | X | X |  |  | X |  |  |  |  |  |  |  |  |  |  |  |  |  |  | X |  |  |  |  |  |  |  |
| Fielding et al., 2009 |  |  |  |  | X | X |  |  | X | X |  |  |  |  |  |  |  |  |  |  |  |  |  | X |  |  | X |  |  |  | X |
| Fielding et al., 2012 |  |  |  |  |  | X |  |  |  |  |  |  |  |  |  |  |  |  |  |  |  |  |  | X |  |  |  |  |  |  | X |
| Kolbe et al., 2014 |  |  |  |  | X | X |  |  | X | X |  |  |  |  |  |  |  |  |  |  |  |  |  |  |  |  | X |  |  |  | X |
| Clough et al., 2015 |  |  |  |  | X | X |  |  |  |  |  |  |  |  |  |  |  |  |  |  |  |  |  | X |  |  | X |  |  |  |  |
| Clough et al., 2015 |  |  |  |  |  | X |  |  |  |  |  |  |  |  |  |  |  |  |  |  |  |  |  |  |  |  |  |  |  |  | X |
| Nygaard et al., 2015 |  |  |  |  |  | X |  |  |  |  |  |  |  |  |  |  |  |  |  |  |  |  |  |  |  |  |  |  |  | X | X |
| de Rodez Benavent et al., 2017 |  |  | X |  | X |  |  |  |  | X |  |  | X |  |  |  |  |  |  |  |  |  |  | X |  |  |  |  | X |  | X |
| Ferreira et al., 2018 | X |  |  |  | X |  |  | X |  |  | X |  |  | X | X | X |  |  |  |  |  |  |  | X |  |  |  |  |  |  |  |
| Gajamange et al., 2019 |  |  |  |  | x | x |  |  |  |  |  |  |  |  |  |  |  |  |  |  |  |  |  |  |  |  |  |  |  |  |  |
| Pavisian et al., 2019 |  |  |  |  | X |  |  |  |  |  |  |  |  |  |  |  |  |  |  |  |  |  | X |  |  | X |  |  |  |  | X |
| Ternes et al., 2019 |  |  |  |  |  |  |  |  |  |  | X |  |  |  |  |  |  |  |  |  |  |  |  |  |  |  |  |  |  |  |  |
| Zangemeister et al., 2020 |  | X |  |  | X | X |  |  |  |  |  |  |  |  |  |  | X |  |  |  |  |  |  | X | X |  |  | X | X | X | X |
| Nij Bijvank et al., 2021 |  |  |  | X |  |  |  |  |  |  | X |  |  |  |  |  |  | X | X |  |  |  |  |  |  |  |  |  |  |  | X |
| Gehrig et al., 2022 |  |  |  |  | X |  |  |  |  |  |  |  |  |  |  |  |  |  |  |  |  |  |  |  |  |  |  |  |  | X | X |
| Nij Bijvank et al., 2023 |  |  |  | X |  |  |  |  |  |  | X |  |  |  |  |  |  | X | X |  |  |  |  |  |  |  |  |  |  |  | X |
| de Villers-Sidani et al., 2023 |  |  | X |  | X |  | X |  |  |  |  |  | X |  |  |  |  |  |  |  |  |  |  |  |  |  |  | X |  | X | X |
| Polet et al., 2023 |  |  |  |  |  |  |  |  |  |  |  | X |  |  |  |  |  |  |  | X | X | X |  |  |  |  |  |  |  |  |  |
|  | MOCA, Montreal Cognitive Assessment; MACFIMS, Minimal Assessment of Cognitive Function in MS; BICAMS, Brief International Cognitive Assessment for MS; BRBNT, Brief Repeatable Battery of Neuropsychological Tests; SDMT, Symbol Digit Modalities Test; PASAT, Paced Auditory Serial Addition Test; AVLT, Auditory Verbal Learning Test; LWF, Learning Without Forgetting; SR, The Sentence Repetition test; RAVLT, Rey Auditory Verbal Learning Test; DIGIT SPAN FORW, Digit Span Forward; DIGIT SPAN BACK, Digit Span Backward; CVLT, The Californian Verbal Learning Test; STROOP, The Stroop Test; CORSI BLOCK TEST, The Corsi block-tapping task; BENTON FRT, the Benton Facial Recognition Test; WISCONSIN CST, The Wisconsin Card Sorting Test; BVMT-R, Brief Visuospatial Memory Test-Revised; TRAIL MAKING, The Trail Making Test; COWAT, Controlled Oral Word Association Test; DKEFS 20Q, the Delis-Kaplan Executive Function System test; TAP, Test of Attentional Performance; CONCEPT SHIFTING TEST, The Concept Shifting Test; BEC-96, Batterie d'évaluation cognitive; HADS, Hospital Anxiety and Depression Scale; BDI, Beck Depression Inventory; HAQUAMS, Hamburg Quality of Life Questionnaire in Multiple Sclerosis; WTAR, The Wechsler Test of Adult Reading; NART, National Adult Reading Test; MSFC, Multiple Sclerosis Functional Composite; MFIS, Modified Fatigue Impact Scale; FSS, Fatigue Severity Scale; 9HPT, 9-hole pegboard test; EDSS, Expanded Disability Status Scale. | | | | | | | | | | | | | | | | | | | | | | | | | | | | | | |
|  |  |  |  |  |  |  |  |  |  |  |  |  |  |  |  |  |  |  |  |  |  |  |  |  |  |  |  |  |  |  |  |
