# Supplementary material for: Eye-Tracking Metrics as a Digital Biomarker for Neurocognitive Disorders in Multiple Sclerosis: A Scoping Review
Source: Brain Sci. 2025 Jan 31;15(2):149. doi: 10.3390/brainsci15020149 (PMC11852410; doi:10.3390/brainsci15020149)
Supplement: Supplementary file 1 [file brainsci-15-00149-s001.zip › Table S4. Eye-Tracking set up.docx]

| \|  \| Table S4. Eye-Tracking set up \| \| \| \| \| \|  \|  \|  \|  \|  \|  \|  \| \| --- \| --- \| --- \| --- \| --- \| --- \| --- \| --- \| --- \| --- \| --- \| --- \| --- \| --- \| \| **Authors, Published Year** \| **Equipment** \| \| **Type** \| **Sampling Rate** \| **Spatial accuracy** \| **Lighting condition** \| **System recorded** \| **Head movement restrictions** \| **Screen resolution** \| **Screen distance** \| **Calibration** \| **Software for stimula** \| **Software for analysis** \| \| Fielding et al., 2009 \| IRIS infrared eye tracker (Skalar Medical, BV, Delft, The Netherlands) \| \| Screen based \| 1000 Hz \| N/A \| Darkened room \| N/A \| Bite bar \| N/A \| 84 cm \| N/A \| E-Prime software (Psychology Software Tools, Inc, PA, USA) \| Matlab software (Mathworks, Inc., Natick, MA) \| \| Fielding et al., 2009 \| IRIS infrared eye tracker (Skalar Medical, BV, Delft, The Netherlands) \| \| Screen based \| 1000 Hz \| N/A \| Darkened room \| N/A \| Bite bar \| N/A \| 84 cm \| N/A \| E-Prime software (Psychology Software Tools, Inc, PA, USA) \| N/A \| \| Fielding et al., 2012 \| IRIS infrared eye tracker (Skalar Medical, BV, Delft, The Netherlands) \| \| Screen based \| 1000 Hz \| N/A \| N/A \| N/A \| Bite bar \| N/A \| 84 cm \| N/A \| E-Prime software (Psychology Software Tools, Inc, PA, USA) \| N/A \| \| Kolbe et al., 2014 \| IRIS infrared eye tracker (Skalar Medical, BV, Delft, The Netherlands) \| \| Screen based \| 1000 Hz \| N/A \| N/A \| N/A \| Bite bar \| N/A \| 84 cm \| N/A \| E-Prime software (Psychology Software Tools, Inc, PA, USA) \| N/A \| \| Clough et al., 2015 \| EyeLink II dark pupil (SR-Research Ltd, Mississauga, Canada) \| \| Screen based \| 500 Hz \| 0.5° \| N/A \| Monocular \| N/A \| N/A \| 84 cm \| N/A \| Experiment Builder software (SR-Research Ltd, Mississauga, Canada) \| N/A \| \| Clough et al., 2015 \| EyeLink II dark pupil (SR-Research Ltd, Mississauga, Canada) \| \| Screen based \| 500 Hz \| 0.5° \| N/A \| Monocular \| N/A \| N/A \| 84 cm \| N/A \| Experiment Builder software (SR-Research Ltd, Mississauga, Canada) \| N/A \| \| Nygaard et al., 2015 \| iView X Hi-Speed eye-tracking (SensoMotoric Instruments, Teltow, Germany) \| \| Screen based \| 60 Hz \| <0.4° \| N/A \| Binocular \| N/A \| 1680 x 1050 pixels \| 70 cm \| N/A \| N/A \| N/A \| \| de Rodez Benavent et al., 2017 \| SMI RED (SMIGmb, Teltow, Germany) \| \| Screen based \| 60 Hz \| N/A \| Lit with approximately 180 lux \| Binocular \| N/A \| 1680 x 1050 pixels \| 70 cm \| 5 point \| I-View Software (SMIGmb, Teltow, Germany) \| Language C++ \| \| Ferreira et al., 2018 \| SMI RED250 (SMIGmb, Teltow, Germany) \| \| Screen based \| 250 Hz \| <0.4° \| Dimly lit room (∼10 lux) \|  \| Headband \| 1680 x 1050 pixels \| 70 cm \| 5 point \| iView X Software (SMIGmb, Teltow, Germany) \| Matlab software (Mathworks, Inc., Natick, MA), Psycho-physics Toolbox \| \| Gajamange et al., 2019 \| EyeLink 1000, MR-compatible in-frared video eye tracking system \| \| Screen based \| 500 Hz \| N/A \| N/A \| N/A \| N/A \| N/A \| N/A \| N/A \| Matlab software (Mathworks, Inc., Natick, MA) \| Matlab software (Mathworks, Inc., Natick, MA) \| \| Pavisian et al., 2019 \| Gazepoint GP3 HD \| \| Screen based \| 150 Hz \| 0.5°–1.0° \| N/A \| Binocular \| Chin rest \| 1280 x 1024 pixels \| N/A \| 9 point \| Gazepoint API (Application Program Interface) \| OGAMA software (Open Gaze and Mouse Analyzer) \| \| Ternes et al., 2019 \| EyeLink II dark pupil (SR-Research Ltd, Mississauga, Canada) \| \| Screen based \| 500 Hz \| 0.5° \| N/A \| Monocular \| N/A \| N/A \| 84 cm \| N/A \| Experiment Builder software (SR-Research Ltd, Mississauga, Canada) \| N/A \| \| Zangemeister et al., 2020 \| OBER JazzNovo \| \| Screen based \| 1000 Hz \| <0.1° \| N/A \| N/A \| Forehead support \| N/A \| 57 cm \| 9 point \| Eye Track Project Software \| N/A \| \| Nij Bijvank et al., 2021 \| EyeLink 1000 Plus (SR-Research Ltd, Mississauga, Canada) \| \| Screen based \| 1000 Hz \| 0.25°- 0.50° \| 20 to 50 Lux \| N/A \| Chin and a forehead rest \| 1024 x 768 pixels \| 50–55 cm \| 9 point \| N/A \| Matlab software (Mathworks, Inc., Natick, MA) \| \| Gehrig et al., 2022 \| Tobii Pro X2-60 (Tobii Technology, Stockholm, Sweden) \| \| Screen based \| 60 Hz \| N/A \| Standardized room lighting \| Binocular \| N/A \| 1.920 x 1.080 pixels \| N/A \| 9 point \| Tobii Pro Studio (Tobii Technology, Stockholm, Sweden) \| Tobii Pro Studio (Tobii Technology, Stockholm, Sweden) and Matlab software (Mathworks, Inc., Natick, MA) \| \| Nij Bijvank et al., 2023 \| EyeLink 1000 Plus (SR-Research Ltd, Mississauga, Canada) \| \| Screen based \| 1000 Hz \| 0.25°- 0.50° \| 20 to 50 Lux \| Binocular \| Chin and a forehead rest \| 1024 x 768 pixels \| 50–55 cm \| 9 point \| N/A \| Matlab software (Mathworks, Inc., Natick, MA) \| \| de Villers-Sidani et al., 2023 \| ETNA™ Ipad Pro tablet \| \| Tablet based \| 60 Hz \| 0.47° \| N/A \| N/A \| N/A \| N/A \| N/A \| N/A \| ETNA™ software \| ETNA™ software \| \| Polet et al., 2023 \| Mobile Eye-BrainT2, SURICOG \| \| Screen based \| 300 Hz \| 0.25° \| N/A \| N/A \| Head-mounted \| N/A \| 60 cm \| N/A \| N/A \| N/A \| \|  \| \| N/A = Not Available \| \| \| \|  \|  \|  \|  \|  \|  \|  \|  \| |
| --- | --- | --- | --- | --- | --- | --- | --- | --- | --- | --- | --- | --- | --- | --- | --- | --- | --- | --- | --- | --- | --- | --- | --- | --- | --- | --- | --- | --- | --- | --- | --- | --- | --- | --- | --- | --- | --- | --- | --- | --- | --- | --- | --- | --- | --- | --- | --- | --- | --- | --- | --- | --- | --- | --- | --- | --- | --- | --- | --- | --- | --- | --- | --- | --- | --- | --- | --- | --- | --- | --- | --- | --- | --- | --- | --- | --- | --- | --- | --- | --- | --- | --- | --- | --- | --- | --- | --- | --- | --- | --- | --- | --- | --- | --- | --- | --- | --- | --- | --- | --- | --- | --- | --- | --- | --- | --- | --- | --- | --- | --- | --- | --- | --- | --- | --- | --- | --- | --- | --- | --- | --- | --- | --- | --- | --- | --- | --- | --- | --- | --- | --- | --- | --- | --- | --- | --- | --- | --- | --- | --- | --- | --- | --- | --- | --- | --- | --- | --- | --- | --- | --- | --- | --- | --- | --- | --- | --- | --- | --- | --- | --- | --- | --- | --- | --- | --- | --- | --- | --- | --- | --- | --- | --- | --- | --- | --- | --- | --- | --- | --- | --- | --- | --- | --- | --- | --- | --- | --- | --- | --- | --- | --- | --- | --- | --- | --- | --- | --- | --- | --- | --- | --- | --- | --- | --- | --- | --- | --- | --- | --- | --- | --- | --- | --- | --- | --- | --- | --- | --- | --- | --- | --- | --- | --- | --- | --- | --- | --- | --- | --- | --- | --- | --- | --- | --- | --- | --- | --- | --- | --- | --- | --- | --- | --- | --- | --- | --- | --- | --- | --- | --- | --- | --- | --- | --- | --- | --- | --- | --- | --- | --- | --- | --- | --- | --- | --- | --- | --- | --- | --- | --- | --- | --- | --- | --- | --- | --- | --- | --- | --- | --- | --- | --- | --- | --- | --- | --- | --- | --- | --- | --- | --- | --- | --- |
