# Supplementary material for: Eye-Tracking Metrics as a Digital Biomarker for Neurocognitive Disorders in Multiple Sclerosis: A Scoping Review
Source: Brain Sci. 2025 Jan 31;15(2):149. doi: 10.3390/brainsci15020149 (PMC11852410; doi:10.3390/brainsci15020149)
Supplement: Supplementary file 1 [file brainsci-15-00149-s001.zip › Table S5. Experimental paradigms, metrics and results.docx]

| \| Table S5. Experimental paradigms, metrics and results \| \| \| \| \| --- \| --- \| --- \| --- \| \| **Authors, Published Year** \| **Experimental paradigms** \| **Metrics** \| **Results (Correlation between Cognitive assessment and Eye movements)** \| \| Fielding et al., 2009 \| Unpredictable visually guided saccades Predictable visually guided saccades Endogenously cued visually guided saccades \| LATENCY, FINAL EYE POSITION (GAIN), ABSOLUTE POSITION ERROR (ABSerror), ERRORS (PROPORTION) \| ABSerror for unpredictable saccades in the presence of distractors correlated significantly with Digit Span scores (r = 0.60, p = 0.003). FEP gain for predictable saccades in the absence of distractors correlated significantly with PASAT scores (r = 0.544, p < 0.01). PASAT scores also correlated with ABS error after valid cues (r = -0.523, p < 0.025), and with variability of ABS error (r = -0.568, p < 0.01). \| \| Fielding et al., 2009 \| Antisaccade task \| LATENCY, ACCURACY \| A significant correlation was found between proportion of errors and scores on the PASAT (r = -.57, p = 005) for persons with MS, demonstrating an inverse relationship between the number of errors made on this task and the PASAT. No other significant correlations were found, including those between EDSS or BDI scores and any of the other experimental measures. \| \| Fielding et al., 2012 \| Antisaccade task \| AS ERRORS (%), AS LATENCY (ms), AS POSITION ERROR (%) \| In persons with MS, significant correlations were found between PASAT scores (% correct) and AS error rate (r = -.54, p < 0.01) and AS position error (r = -.54, p < 0.01). In a two-year follow-up study of RRMS patients, a number of saccade parameters were significantly altered compared to controls, particularly those proposed to reflect attentional and working memory processes. Importantly, these changes were evident in the absence of clinically apparent changes in functional status, and associations with neuropsychological assessments were maintained over time. In particular, measures of error and accuracy remained associated with scores on the PASAT. \| \| Kolbe et al., 2014 \| Antisaccade task \| AS ERRORS (%), AS LATENCY (ms) \| Patients scored lower on the PASAT (P = 0.02) and SDMT (P < 0.0001), and reductions in scores on both tests were associated with a greater number of AS errors (PASAT: q 5 20.48, P = 0.03; SDMT: q 5 20.66, P = 0.001). There were no significant correlations between clinical or cognitive variables and AS latency. \| \| Clough et al., 2015 \| Ocular motor WMem task \| LATENCY, WMEM ERRORS, WMEM EFFECT \| Patients with late stage CDMS had a significantly greater WMem effect (and therefore poorer WMem capacity) compared to controls; no significant differences were found between controls and CIS or controls and early stage CDMS patients. In all patient groups, the WMem effect increased with increasing disease duration. A larger WMem effect, and thus poorer WMem capacity, was significantly associated with poorer PASAT performance (controls only) and SDMT performance (controls and CDMS late patients). No relationship was found between WMem effect and any of the neuropsychological tests in CIS or early CDMS patients. \| \| Clough et al., 2015 \| Visually guided saccades Antisaccades Memory-guided saccades Endogenously cued saccades \| LATENCY, ERRORS \| In the visually guided saccade task for CDMS late patients only, longer latencies were significantly related to poorer PASAT performance. In the antisaccade task poorer PASAT performance was significantly associated with a higher proportion of errors (controls, CDMS early and CDMS late), longer error correction times (CIS and CDMS late) and longer saccade latencies (CDMS late). In the memory-guided saccade task, longer saccade latencies were significantly associated with poorer PASAT performance only in patients with CDMS late. In the endogenously cued saccade task for controls only, a higher proportion of errors was associated with poorer PASAT performance. \| \| Nygaard et al., 2015 \| Saccadic task \| SACCADIC INITIATION TIME, TIME TO TARGET AOI, NUMBER OF VALID SACCADES \| Patients' SI time was associated with wSDMT but not with PASAT. In the healthy controls, we found no association between SI time and wSDMT (r = -0.189, p = 0.256). \| \| de Rodez Benavent et al., 2017 \| Pupillometric experiment of cognitive load \| PUPILLARY DILATION \| Patients and controls had similar pupil dilation curves during problem solving. Regression analyses showed that processing speed (SDMT) scores were not associated with pupil dilation in the participants in general (r = -.002, p = .985) or in the MS patient group alone (r = -.075, p = .690). They also found no correlation between verbal memory (CVLT) and pupil dilation, either in the group as a whole (r = 0.060, p = .599) or in the patients (r = -.007, p = .966). There was also no correlation between performance on the visuospatial tests and pupil dilation in either all participants (r = 0.171, p = .131) or patients (r = 0.261, p = .108). \| \| Ferreira et al., 2018 \| Antisaccade paradigm and position traces of the eyes \| AS ERRORS, AS LATENCY, AS PEAK-VELOCITY \| There were no statistically significant correlations between scores on all other neuropsychological tests, AS errors and latency. \| \| Gajamange et al., 2019 \| Prosaccade and antisaccade ocular motor paradigm \| LATENCY, DIRECTIONAL ERROR RATE \| Correlation between cognitive ocular motor and neuropsychological variables in patients: antisaccade latency correlated with PASAT (ρ = -0.75, p = 0.47 x 10^-3^) and AS directional error rate correlated with SDMT scores (ρ = -0.48, p = 0.043). PS latency correlated with AS latency (r = 0.83, p = 0.02 × 10^-3^) and PS error rate correlated with AS error rate (ρ = 0.81, p = 0.04 × 10^-3^). \| \| Pavisian et al., 2019 \| SDMT \| TOTAL MEAN SACCADE DISTANCE IN THE KEY AREA, NUMBER OF VISITS TO KEY AREA PER RESPONSE, NUMBER OF FIXATIONS IN THE TEST AREA \| People with MS perform slower on the SDMT than healthy individuals and eye movement metrics are predictive of performance. The number of key area visits per response was shown to be the strongest predictor of SDMT performance (F = 55.212; p < 0.001), accounting for 54.9% of its variance. \| \| Ternes et al., 2019 \| Antisaccade task Endogenously cued saccade task \| AS ERRORS, ENDOGENOUSLY CUED SACCADE ERRORS \| Analyses showed that compared to the control group, MS participants made significantly more antisaccade errors and had a lower Stroop interference ratio. There was no difference between the groups in the number of endogenously cued saccade errors. \| \| Zangemeister et al., 2020 \| TAP Alertness \| PEAK ACCELERATION SACCADES, PEAK VELOCITY SACCADES, AMPLITUDE SACCADES, DURATION SACCADES \| The correlation of dynamic parameters, i.e. peak positive acceleration and peak positive velocity, with both MFIS Cog and Nine-HPT is highly significant, compared with amplitude and duration, which are less valuable in this regard. \| \| Nij Bijvank et al., 2021 \| Pro-saccadic task Anti-saccadic task \| LATENCY, PROPORTION ERRORS AS, GAIN PS \| Higher latency of 15 degree saccade was associated with poorer executive function (β = -0.49, p < .001), information processing speed (β = -0.37, p < .001) and working memory (β = -0.36, p < .001). The latency of 8 degree saccades and the latency of correct antisaccades showed similar relationships. A longer correction latency in the anti-saccade task was associated with poorer attention (β = -0.22, p = .015). More errors were associated with lower scores in five domains, most strongly in information processing speed (β = -0.36, p < .001) and attention (β = -0.31, p = .001). Peak velocity, Pv/Am and gain of pro-saccades were not strongly related to clinical and cognitive function. \| \| Gehrig et al., 2022 \| Visual search in naturalistic scenes \| “TIME TO FIRST FIXATION” (TFF), “FIXATIONS BEFORE” (FB), “TOTAL FIXATION DURATION” (TFD), “FIXATION COUNT” (FC) FOR THE AOI \| In the exploratory analyses of the visual search behaviour of persons with MS, this study found significant effects of their SDMT performance (SDMTz>-1 vs. SDMTz<-1) on TFF (p = 0.006),TFD (p = 0.005) and FC (p = 0.033). Patients with average or better SDMT performance had a faster first fixation (1.55s ± 0.40s vs. 1.96s ± 0.33s), more (6.08s ± 1.48s vs. 4.71s ± 2.05s) and longer (2.47s ± 1.19s vs. 1.29s ± 0.71s). Patients with MS took longer to find and fixate the target object, and although they took longer, they were less accurate. They fixated the target for less time. The authors also found significant correlations between SDMT performance and TFF expected (r = -0.498, p = 0.003) but not TFF unexpected (r = -0.212, p = 0.229), and between SDMT and TFD expected (r = 0.441, p = 0.009) and TFD unexpected (r = 0.484, p = 0.004). \| \| Nij Bijvank et al., 2023 \| The double-step saccadic task \| PROPORTION OF CORRECT DOUBLE-STEP SACCADES, DIRECTION DIFFERENCE FIRST SACCADE, ABSOLUTE ERROR FEP, HORIZONTAL ERROR FEP, VERTICAL ERROR FEP \| Cognitively impaired individuals with multiple sclerosis showed a lower proportion of correct responses (B = -0.11, P = 0.002) and acceptable responses (B = -0.11, P = 0.003), a higher proportion of first saccades directed to the second target location (B = 0.04, P = 0.046), a larger direction difference from the first saccade (B = 3. 8, P = 0.016), a larger absolute error of the final eye position (B = 0.65, P < 0.001), a more negative horizontal error of the final eye position (B = -0.31, P = 0.005), a more negative vertical error of the final eye position (B = -0.86, P < 0.001) and a larger direction difference from the first saccade (B = 3.8, P = 0.017). When the associations of these parameters with the z-scores of individual cognitive domains were examined, strong significant associations were found with all cognitive domains except verbal fluency. \| \| de Villers-Sidani et al., 2023 \| Fixation task Pro-saccade task Anti-saccade task Smooth pursuit task \| OSI, LATENCY, FIRST SACCADE GAIN, PEAK VELOCITY, AS CORRECT, PURSUIT VELOCITY GAIN \| Nine eye movement parameters were significantly correlated with the SDMT, five with the BICAMS, ten with the MSFC and nine with the EDSS. A greater percentage of pro-saccadic (56%) and anti-saccadic (44%) parameters were significantly correlated with MS-related clinical scale scores than fixation (25%) and smooth pursuit (6%) parameters. \| \| Polet et al., 2023 \| Facial emotion recognition \| NUMBER OF FIXATIONS, AVERAGE FIXATION DURATION \| There was no difference between groups in the number of fixations during FER (p = 0.29). The SPMS group had longer fixation times on the face than the HC and RIS groups (all p < 0.05). The SPMS group had longer fixation times than the HC and RIS groups on the face for anger and neutral recognition (all p < 0.05) and on the mouth for fear recognition (all p < 0.05). Compared to the HC group, the SPMS group had a longer fixation time on the face for happiness and surprise recognition (all p = 0.05) and on the mouth for neutral condition recognition (p = 0.05). The SPMS group also spent more time fixating the nose than the RIS group for anger recognition (p< 0.05). Finally, the SPMS group spent more time looking at the face than the CIS group for happiness recognition (p = 0.05). The HC group made more fixations on the nose than the CIS group for surprise recognition (p < 0.05). There was no significant difference in eye behaviour between the groups for disgust and sadness recognition. \| \| ABSerror, Absolute position error; BDI, Beck Depression Inventory; BICAMS, Brief International Cognitive Assessment for MS, FEB, Final Eye Position; CDMS, Clinically Definite MS; CVLT, The California Verbal Learning Test; EDSS, Expanded Disability Status Scale; FB, Fixations Before; FC, Fixation Count; MFIS Cog, Modified Fatigue Impact Scale – Cognition; Nine-HPT, Nine-Hole-Peg-Test; PASAT, Paced Auditory Serial Addition Task; SDMT, The Symbol Digit Modalities Test; TFD, Total Fixation Duration; TFF, Time to First Fixation; WM, Working Memory. \| \| \| \| |
| --- | --- | --- | --- | --- | --- | --- | --- | --- | --- | --- | --- | --- | --- | --- | --- | --- | --- | --- | --- | --- | --- | --- | --- | --- | --- | --- | --- | --- | --- | --- | --- | --- | --- | --- | --- | --- | --- | --- | --- | --- | --- | --- | --- | --- | --- | --- | --- | --- | --- | --- | --- | --- | --- | --- | --- | --- | --- | --- | --- | --- | --- | --- | --- | --- | --- | --- | --- | --- | --- | --- | --- | --- | --- | --- | --- | --- | --- | --- | --- | --- | --- | --- | --- | --- |
